# HCV/HIV coinfection among people who inject drugs and enter opioid substitution treatment in Greece: prevalence and correlates

**DOI:** 10.1186/s41124-016-0017-5

**Published:** 2016-08-25

**Authors:** Anastasios Fotiou, Eleftheria Kanavou, Argyro Antaraki, Clive Richardson, Manina Terzidou, Anna Kokkevi, Drug Related Infectious Diseases (DRID) Medical Doctors Group of OKANA, Konstantina Alexakou, Olga Anagnostou, Giorgos Androulakis, Emmanuel Chalkiadakis, Ioanna Detsi, Anastasia Drimousi, Dimitra Frontzou, Melpomeni Gargoulaki, Maria Iosifidou, Katerina Kaliva, Anna Katsilli, Stavroula Kollia, Maria Kollitsida, Argyrios Kotsalis, Spiroula Makristathi, Theodoros Papadopoulos, Eleftheria Petroulaki, Vasilis Pinakas, Michael Spirellis, Konstantinos Stamatopoulos, Victoria Stavridou, Yolanda-Irena Stepen-Topalidi, Christos Tanis, Anastasios Tsantilas, Panayiota Tsekoura, Efrosini Tsirogianni

**Affiliations:** 1 Epidemiology Unit, Greek Reitox Focal Point of the EMCDDA, University Mental Health Research Institute, 2 Soranou tou Efesiou St., Athens, 11527 Greece; 2 Panteion University of Social and Political Sciences, 136, Leoforos A. Siggrou, Kallithea, Athens 17671 Greece; 3 University Mental Health Research Institute, 2 Soranou tou Efesiou St., Athens, 11527 Greece; 4 Greek Organisation Against Drugs, Athens Greece, 21, Averof st., 10433 Athens, Greece

**Keywords:** Hepatitis C virus, Human immunodeficiency virus, HCV/HIV coinfection, People who inject drugs, PWID, Opioid substitution treatment, Risk factors, Greece

## Abstract

**Background:**

HCV/HIV coinfection in people who inject drugs is a public health issue, which presents a variety of challenges to healthcare providers. The determinants of HCV/HIV coinfection in this population are nonetheless not well known. The aim of the present study is to identify the factors associated with HCV/HIV coinfection in people who inject drugs and enter drug-related treatment.

**Methods:**

Linked serological and behavioral data were collected from people who entered 38 opioid substitution treatment clinics in central and southern Greece between January and December 2013. Three mutually exclusive groups were defined based on the presence of HCV and HIV antibodies. Group 1 clients had neither infection, Group 2 had HCV but not HIV, and Group 3 had HCV/HIV coinfection. Multinomial logistic regression analyses identified differences between groups according to socio-demographic, drug use and higher-risk behavioral characteristics.

**Results:**

Our study population consisted of 580 people who injected drugs in the past 12 months (79.8 % males, with median age 36 years).79.4 % were HCV and 15.7 % HIV infected. Of those with complete serological data in both HCV and HIV indicators, 20.4 % were uninfected, 64.0 % HCV monoinfected, and 14.9 % HCV/HIV coinfected. HCV infection with or without HIV coinfection was positively associated with living alone or with a spouse/partner without children, prior incarceration, drug injecting histories of ≥10 years, and syringe sharing in the past 12 months, and negatively associated with never having previously been tested for HCV. HCV/HIV coinfection, but not HCV infection alone, was positively associated with residence in urban areas (relative risk ratio [RRR] = 4.8, 95 % confidence interval [CI]: 1.7–13.7, *p* = 0.004) and averaging >3 injections a day in the past 30 days (RRR = 4.5, 95 % CI: 1.6–12.8, *p* = 0.005), and negatively associated with using a condom in the last sexual intercourse.

**Conclusions:**

People who inject drugs and live in urban areas and inject frequently have higher risk of coinfection. Findings highlight the need for scaling-up needle and syringe programs in inner city areas and promoting access of this population to screening and treatment, especially in prisons. The protective role of living with parents and children could inform the implementation of indicated interventions.

## Background

Intravenous drug use is responsible for the majority of new HCV infections and HCV is the most prevalent viral infection among people who inject drugs [1–3]. It is estimated that about 44 % of current injection drug users in the countries of the European Union (EU) and the European Free Trade Association (EFTA) have HCV RNA [4]. HIV often co-occurs with HCV as both are transmitted through infected blood, mainly through injection drug use [1, 2, 5–7]. Chronic HCV infection is the most common comorbidity in HIV infected drug users, with the prevalence of HCV infection among HIV infected drug users reaching rates higher than 70 % in several countries and regions in western [1, 8] and eastern Europe [1], Latin America and the Caribbean [1], and Asia [1, 7, 8].

HCV/HIV coinfection in drug users is a growing public health concern. While HCV infection in itself causes substantial morbidity and mortality [9–11], patients with HIV co-infection have much higher odds to accelerate HCV infection to endstage liver disease than those infected with hepatitis C alone [8, 12–14]. Coinfection with HIV also contributes to development or acceleration of cardiovascular disease, neurocognitive impairment, insulin resistance, and renal insufficiency [15].

HCV/HIV coinfection presents a variety of challenges to healthcare providers [16–19]. HIV/HCV coinfected drug users have significantly higher risk of having poorer physical and mental health and use more healthcare services compared to those infected with HIV only [20, 21]. Co-occurring HCV decreases the benefits of antiretroviral therapy (ART) [10], while drug addiction complicates treatment regimens and leads to treatment-related adverse events [22]. HIV/HCV coinfected people with long injection histories are significantly less likely to achieve virologic suppression and CD4 cell count recovery, and have a higher risk of death [16, 23]. Finally, coinfected drug users do not have equitable and universal access to HIV/AIDS and HCV treatment [16, 24, 25]. Barriers to treatment may be aggravated by the stigma associated with drug users with comorbidities, and preconceptions shared among healthcare providers who assume low compliance, high risk of reinfection, and poor response to ART [26, 27].

Given the evidence suggesting higher morbidity and mortality among coinfected drug users, there is a need to document the prevalence of coinfection in this population and to identify factors that put HCV uninfected or HCV monoinfected populations at risk for HIV infection. The rather limited evidence available points to the independent role of various behavioral and socio-demographic factors. Behavioral factors include: longer periods of injection drug use [5, 28–33]; receptive sharing of injection equipment [5, 32–36]; frequent injection [5, 31–33, 35]; present or past incarceration [29, 35, 37, 38]; drug use during incarceration [39]; and high risk sexual behavior [30]. Socio-demographic factors include: residence in metropolitan areas where injection drug use takes place [5]; female gender [5]; older age [5, 39]; ethnicity (in northern American studies, e.g., Hispanic in [29] and Canadian aboriginal in [5]); and lower education [28].

In Greece an estimated 2.0–2.6 people per 1000 people aged 15–64 years are heroin drug users [40, 41]. Problems with heroin or other opioids are reported by the majority (69 %) of people entering treatment [41]. In 2014, the estimated prevalence of antibodies to HCV among injection drugs users entering all types of drug-related treatment was 71 % overall, and 80 % among first-ever treatment entries [41]. Until 2011, HIV among injection drug users never exceeded 3 % of new HIV diagnoses reported annually [41–43]. In 2011, the number of new HIV diagnoses increased sharply, rising from 5.5 in 2010 to 10.7 per 100,000 in 2012 [44, 45]. These increases were observed only in Athens and were driven by injecting drug use [42, 44, 45]. Studies conducted in community samples of injection drug users in Athens suggested an HIV prevalence of up to about 15 % [46].

In Greece, little is known about the profiles of injection drugs users characterized by different infection statuses for HCV and HIV (e.g., [43, 47, 48]), while no published study is known to the authors to have explored the factors associated with coinfection. The present study attempts to fill this gap and aims at identifying injection drug users in the country who are at the greatest infection risk based on their sociodemographic, drug use and higher-risk behavioral characteristics. More specifically, drawing from existing knowledge, the study aims to differentiate drug users at risk of HCV monoinfection from those at risk of HCV/HIV coinfection so that policy makers and health care and harm reduction practitioners working close to this population could utilize findings to design appropriate prevention programs and help drug users with differing needs.

## Methods

### Study population and process

The study population consisted of all heroin or other opioid users who started treatment in outpatient opioid substitution treatment (OST) clinics of the Greek Organization Against Drugs (OKANA) in central and southern Greece in the period between January 1 and December 31, 2013 and had injected drugs in the 12 months preceding their entry (*n* = 580). Given the study aims, the inclusion criteria were dictated solely by the need to have data on a wide range of behavioral variables that could be used as explanatory variables and therefore allow for more meaningful analyses. During the data collection period (2013) only the OST outpatient clinics located in southern and central Greece had established a data collection system which included a wide range of behavioral variables. The 38 clinics from which the participants were recruited had similar organisational structure and employed common treatment protocols. They were located in the capital cities of 14 prefectures in 9 of the 13 administrative regions of the country (Attika, Peloponnese, Crete, Epirus, Thessaly, Western Greece, Central Greece, Ionian Islands, and the Southern Aegean region; representing about 73 % of the total population in Greece). OST clinics and participants in the study formed about 70 % of the total OST outpatient clinics in operation and 72 % of the past 12 month injection drug users who entered OST in the country in 2013.

Serological and behavioral data, linked through an anonymised identification code, were collected in the clinics through a routine data collection system established by the Greek REITOX Focal Point of the European Monitoring Centre for Drugs and Drug Addiction (EMCDDA) (henceforth, Focal Point) since the end of the 1990s. Data collection was paper-based and involved all drug users starting treatment in the clinics. Upon entry, each person was tested for HCV and HIV antibodies and interviewed by health practitioners working in the respective clinic. Interviews were guided by the use of a standardised structured questionnaire. Serological testing was provided for by internal OST treatment protocols. No client-level refusals were reported (although there were questionnaires with incomplete serological data, attributed to reasons other than refusing to test or to report results). Completed questionnaires were subsequently sent to the Focal Point, where they were checked for incomplete data and inconsistencies. The questionnaires were scanned and entered in the electronic database. A second round of (electronic) checks for data entry errors, excessive number of missing values, logical inconsistencies, and double-counting was conducted by an in-house statistician. The collection and management of the data at the Focal Point was approved by the Hellenic Data Protection Authority (Decision number: 2186, 1/11/2001).

### Measures

The outcome of interest was HCV and HIV infection status as a proxy of infection risk. Data were based on serological tests (blood samples). Antibodies to HCV, indicating HCV exposure, were detected by enzyme-linked immunosorbent assay (ELISA), with recombinant immunoblot assay (RIBA) or enzyme immunoassay (EIA) confirmation (in all cases, commercial kits were used). Antibodies to HIV, documenting HIV infection, were detected by ELISA, with Western Blot immunoassay for HIV-1/2 confirmation. The presence of HCV antibodies and documented HIV infection indicated HCV/HIV coinfection, the main outcome variable of the study. All prevalence estimates reported are antibody prevalences.

Explanatory variables used in the analyses are shown in Table 1. These were based on self-reports and comprised socio-demographic, drug use, and higher-risk behavioral indicators recommended by the EMCDDA for monitoring drug-related infectious diseases at the national level [49, 50] and are supported by the relevant literature (reviewed in the Introduction [5, 28–39]).

**Table 1:** Sample characteristics in total sample (*n* = 580) and in groups defined by HIV/HCV infection status (*n* = 541)^a^

	Total sample (*n* = 580)	Uninfected^b^ (*n* = 111, 20.4 %)	HCV monoinfected^c^ (*n* = 349, 64.0 %)	HCV/HIV coinfected^d^ (*n* = 81, 14.9 %)
	*N* (%)^e^	*N* (%)	*N* (%)	*N* (%)
Male (vs. Female)^f^	463 (79.8)	95 (85.6)	272 (77.9)	67 (82.7)
Median age [Inter-quartile range]	36 [12]	35 [7]	36 [14]	33 [10]
Greek nationality (vs. Non-Greek nationality)	554 (95.5)	109 (98.2)	332 (95.1)	76 (93.8)
Lived in urban area in the last 5 years (vs. Semi-urban/rural area in the last 5 years)	444 (79.6)	84 (77.1)	264 (78.8)	71 (91.0)
Living alone or with spouse/partner without children (vs. Living with family^g^)	182 (31.9)	23 (20.7)	120 (34.4)	30 (37.0)
Homeless ≥1 night in the past 12 months (vs. Never in the past 12 months)	186 (32.6)	26 (23.4)	107 (31.2)	42 (53.2)
Did not graduate high school (12^th^ grade) (vs. Graduated high school)	360 (62.1)	61 (55.5)	215 (62.0)	59 (74.7)
Employed (vs. Unemployed/student/other and economically inactive^h^)	137 (23.7)	32 (28.8)	88 (25.4)	9 (11.1)
Incarcerated at least once in lifetime (vs. Never)	382 (66.7)	55 (50.5)	241 (69.3)	57 (72.2)
Use of primary substance ≥ 4 days a week (vs. Less frequently)	466 (80.5)	93 (83.8)	272 (78.2)	71 (87.7)
Use of ≥3 substances of abuse^i^ (vs. <3 substances of abuse)	367 (63.3)	66 (59.5)	219 (62.8)	53 (65.4)
Median length of injection (years) [Inter-quartile range]	14 [12]	10 [11]	15 [12]	13 [12]
Mean times of injection per day in the past 30 days [Standard deviation]	1.9 [3.3]	1.2 [2.0]	1.6 [3.0]	3.9 [5.4]
Non-sterile syringe in last injection (vs. Sterile syringe in last injection)	45 (8.1)	5 (4.8)	25 (7.5)	10 (12.7)
Shared syringes in the past 12 months (vs. No syringe sharing in the past 12 months)	137 (25.4)	16 (15.1)	75 (23.1)	33 (44.6)
Shared other injection equipment in the past 12 months (vs. Never in the past 12 months)	237 (43.7)	42 (40.4)	131 (40.3)	44 (57.1)
Two or more sexual partners in the past 12 months (vs. <2 partners)	211 (36.6)	42 (38.9)	120 (34.5)	35 (43.2)
Sex in exchange for money etc. in the past 12 months (vs. Never in the past 12 months/non-active)	37 (6.5)	4 (3.7)	22 (6.4)	8 (10.1)
No condom use in the last intercourse (vs. Use of condom use or non-active)	246 (43.5)	51 (47.7)	155 (45.2)	21 (26.6)
Ever entered treatment for drug-related problems before (vs. Never before)	388 (67.7)	67 (62.6)	244 (69.9)	51 (63.8)
Never tested for HCV before (vs. Tested for HCV before)	182 (32.1)	56 (51.9)	109 (32.2)	10 (12.3)
Never tested for HIV before (vs. Tested for HIV before)	192 (30.7)	50 (45.9)	100 (29.2)	12 (14.8)

^a^OST entrants with both statuses known, excluding four with HIV monoinfection. ^b^Diagnosed HCV negative and HIV negative. ^c^Diagnosed HCV positive and HIV negative. ^d^Diagnosed HCV positive and HIV positive. ^e^Where applicable, Median [Interquartile means] or Mean [Standard deviation]. ^f^Gender was measured by the following item: “What is your gender?” Response options included “male”, “female”, “other (transgender)”. No person responded “other” in the sample used in the present analysis. ^g^Includes children and/or parents. ^h^The category “economically inactive” includes the long-term sick, unpaid carers and persons living on pensions or benefits, but excludes students. ^i^Tobacco use was not measured. Possible abuse of alcohol or non-medical use of prescription drugs is included

### Statistical analysis

Complete serological and behavioral data were collected for 545 treatment entrants (94.0 %). We were interested in identifying injection drug users at the greatest infection risk from their socio-demographic, drug use and higher-risk behavioral characteristics, as well as differentiating those at risk of HCV monoinfection from those at risk of HCV/HIV coinfection. Multinomial logistic regression analyses were conducted to identify differences between different levels of infection status according to these characteristics. Three mutually exclusive groups of injection drug users were defined based on the presence of HCV and HIV antibodies. Group 1 clients had neither infection (uninfected, *n* = 111), Group 2 had HCV but not HIV (HCV monoinfected, *n* = 349), and Group 3 had HCV/HIV coinfection (*n* = 81). These groups formed the three levels of infection status. A fourth group – those with HIV but not HCV (HIV monoinfected) – consisted of only four persons and was excluded from the analyses. Also excluded were 35 cases with missing serological data in either HCV (*n* = 17, of whom 3 were HIV positive) or HIV (*n* = 18, of whom 17 were HCV positive) indicators.

Explanatory variables (all categorical) were first tested in univariate multinomial logistic regression analyses. Variables with *p* < 0.05 and gender were included in the multivariable model. The final regression model included only the variables which were statistically significant (*p* < 0.10) in the multivariable model and was fitted to the data from 450 cases for which complete data were available. Likelihood ratio tests were carried out for the overall effect of an explanatory variable and Wald tests for the coefficients of individual categories against the reference category. Analyses were conducted using IBM SPSS Statistics for Windows, Version 22.0 (Armonk, NY: IBM Corp. IBM Corp. Released 2013). Relative risk ratios (RRR) and 95 % confidence intervals (CI) are presented.

## Results

Table 1 presents socio-demographic, behavioral and serological characteristics of the sample. The vast majority (95.5 %) were of Greek origin, males (79.8 %), with median age 36 years (quartiles: 31, 43) and median length of injection 14 years (quartiles: 8, 20).

HCV infection was detected in 447 (79.4 %) of 563 clients with reported HCV test results and HIV in 88 of 562 cases (15.7 %). The uninfected (Group 1) comprised 20.4 % of those with complete serological data in both HCV and HIV indicators (*n* = 111), 64.0 % were HCV monoinfected (*n* = 349, Group 2) and 14.9 % (*n* = 81) were HCV/HIV coinfected (Group 3). Four people (0.7 %) were HIV monoinfected. The vast majority (95.3 %) of HIV infected people had HCV comorbidity. The HIV/HCV prevalence ratio (i.e., relative prevalence of HIV compared to HCV) was 19.8 %. The prevalence of HIV in HCV positive and HCV negative clients was 18.8 and 3.5 %, respectively (*p* < 0.001).

Among the correlates tested individually for their relation to the infection groups (univariate analyses) a number of factors showed positive association (*p* < 0.05) with infection status. These included: aged ≥35 years, living in an urban area, living alone or with spouse / partner without children, having been homeless in the past 12 months, having not been graduated from high-school, not being employed, having been incarcerated, long injection history, frequent daily injection, and having shared injection equipment in the past 12 months. No condom use in the last intercourse, never having been tested for HCV, and never having been tested for HIV showed negative association with infection status (Table 2).

**Table 2:** Results of univariate analysis of factors associated with HCV monoinfection and HCV/HIV coinfection

		HCV monoinfected^a^ vs. Uninfected^c^				HCV/HIV coinfected^b^ vs. Uninfected				
	*p* ^d^	RRR^e^	95 % CI^f^		*p*	RRR	95 % CI		*p*	*n*
			Lower limit	Upper limit			Lower limit	Upper limit		
Male (vs. Female)^g^	0.164	0.6	0.3	1.1	0.083	0.8	0.4	1.8	0.589	541
Aged ≥35 years (vs. Aged <35 years)	0.015	1.4	0.9	2.2	0.114	0.7	0.4	1.3	0.265	541
Greek nationality (vs. Non-Greek nationality)	0.224	0.4	0.1	1.6	0.174	0.3	0.1	1.5	0.133	541
Lived in urban area in the past 5 years (vs. Lived in semi-urban/rural area)	0.019	1.1	0.7	1.9	0.701	3.0	1.2	7.4	0.016	522
Living alone or with spouse/partner without children (vs. With family^h^)	0.012	2.0	1.2	3.3	0.007	2.3	1.2	4.3	0.013	541
Homeless ≥1 night in the past 12 months (vs. Never in the past 12 months)	<0.001	1.5	0.9	2.4	0.119	3.7	2.0	6.9	<0.001	533
Did not graduate high school (12^th^ grade) (vs. Graduated high school)	0.022	1.3	0.8	2.0	0.225	2.4	1.3	4.5	0.007	536
Unemployed / student / other (vs. Employed)	0.013	1.2	0.7	2.0	0.425	3.0	1.3	6.8	0.009	539
Economically inactive^i^ (vs. Employed)		1.1	0.5	2.2	0.859	4.3	1.6	11.7	0.005	
Incarcerated at least once in lifetime (vs. Never)	0.001	2.2	1.4	3.4	<0.001	2.5	1.4	4.7	0.003	536
Use of primary substance ≥ 4 days a week (vs. <4 days a week)	0.086	0.7	0.4	1.2	0.203	1.4	0.6	3.2	0.454	540
Use of ≥3 substances of abuse (vs. Use of <3 substances of abuse)^j^	0.690	1.1	0.7	1.8	0.534	1.3	0.7	2.3	0.400	541
2–4 years of injection (vs. 0–1 year)	<0.001	1.6	0.5	4.8	0.404	8.8	1.0	78.1	0.051	535
5–9 years of injection (vs. 0–1 year)		2.8	1.0	7.7	0.040	7.8	0.9	66.6	0.060	
≥10 years of injection (vs. 0–1 year)		6.1	2.4	15.1	<0.001	11.0	1.4	87.3	0.023	
Injected on average 2–3 times per day in the past 30 days (vs. <2 times/day)	<0.001	0.7	0.3	1.7	0.472	2.2	0.8	6.1	0.128	529
Injected on average >3 times per day in the past 30 days (vs. <2 times/day)		1.1	0.5	2.4	0.824	5.0	2.1	11.7	<0.001	
Non-sterile syringe in last injection (vs. Sterile syringe in last injection)	0.149	1.6	0.6	4.3	0.342	2.9	0.9	8.9	0.062	519
Shared syringes in the past 12 months (vs. Never in the past 12 months)	<0.001	1.7	0.9	3.1	0.080	4.5	2.2	9.1	<0.001	504
Shared other injection equipment in the past 12 months (vs. Never in the past 12 months)	0.024	1.0	0.6	1.6	0.989	2.0	1.1	3.6	0.026	506
Two or more sexual partners in the past 12 months (vs. <2 sexual partners)	0.300	0.8	0.5	1.3	0.404	1.2	0.7	2.1	0.550	537
Sex in exchange for money etc. in the past 12 months (vs. Never in the past 12 months/non-active)	0.218	1.8	0.6	5.2	0.306	2.9	0.8	10.0	0.092	529
No condom use in last intercourse (vs. Use of condom use or non-active)	0.005	0.9	0.6	1.4	0.654	0.4	0.2	0.7	0.004	529
Ever entered treatment for drug-related problems before (vs. Never before)	0.275	1.4	0.9	2.2	0.157	1.0	0.6	1.9	0.874	536
Never tested for HCV before (vs. Tested for HCV before)	<0.001	0.4	0.3	0.7	<0.001	0.1	0.1	0.3	<0.001	528
Never tested for HIV before (vs. Tested for HIV before)	<0.001	0.5	0.3	0.8	0.001	0.2	0.1	0.4	<0.001	533

^a^Diagnosed HCV positive and HIV negative. ^b^Diagnosed HCV positive and HIV positive. ^c^Diagnosed HCV negative and HIV negative.^d^Overall *p*-value for this covariate. ^e^RRR: relative risk ratios. ^f^95 % confidence intervals. ^g^Gender was measured by the following item: “What is your gender?” Response options included “male”, “female”, “other (transgender)”. No person responded “other” in the sample used in the present analysis.^h^Includes children and or parents. ^i^The category “economically inactive” includes the long-term sick, unpaid carers and persons living on pensions or benefits, but excludes students. In the present analysis, the status “student” was collapsed with the category “unemployed” under the assumption that, like people who are unemployed, students may be motivated to improve their physical and socioeconomic conditions and therefore are ready to undertake fewer health risks. ^j^Tobacco use was not measured. Possible abuse of alcohol or non-medical use of prescription drugs is included

The results of the multivariate analysis (final model comprising only the significant - *p* < 0.10 - variables from the previous model) are shown in Table 3. The probability of belonging to an infected group versus the uninfected group was positively related to living alone or with a spouse/partner without children (*p* = 0.007, overall *p*-value for this covariate), incarceration (*p* = 0.018), having an injecting history of at least 10 years (*p* = 0.002), and having shared syringes in the past 12 months (*p* = 0.002). The risk of infection was reduced in injection drug users who reported that they had never previously been tested for HCV (*p* = 0.001).

**Table 3:** Results of the multinomial logistic regression analysis of factors associated with HCV monoinfection and HCV/HIV coinfection (final model comprising only the significant – *p* < 0.10 – variables from the previous model, *n* = 450)

Correlate (vs. Reference category)		HCV monoinfected^a^ vs. Uninfected^c^				HCV/HIV coinfected^b^ vs. Uninfected			
	*p* ^d^	RRR^e^	95 % CI^f^		*p*	RRR	95 % CI		*p*
			Lower limit	Upper limit			Lower limit	Upper limit	
Male (vs. Female)^g^	0.023	0.4	0.2	0.9	0.018	0.7	0.2	2.0	0.499
Aged ≥35 years (vs. Aged <35 years)	0.015	1.2	0.7	2.1	0.493	0.5	0.2	1.0	0.066
Lived in urban area in the last 5 years (vs. Lived in semi-urban/rural area)	0.006	1.4	0.8	2.6	0.255	4.8	1.7	13.7	0.004
Living alone or with a spouse/partner without children (vs. With family^h^)	0.007	2.6	1.3	4.9	0.005	3.1	1.3	7.3	0.010
Incarcerated at least once in lifetime (vs. Never)	0.018	2.0	1.2	3.4	0.008	2.5	1.1	5.3	0.021
5–9 years of injection (vs. 0–1 year)	0.002	2.4	0.8	7.2	0.134	6.0	0.6	60.6	0.129
2–4 years of injection (vs. 0–1 year)		1.5	0.4	5.6	0.529	8.2	0.7	92.2	0.088
≥10 years of injection (vs. 0–1 year)		5.5	1.9	15.9	0.002	14.1	1.5	133.8	0.021
Injected on average 2–3 times per day in the past 30 days (vs. <2 times/day)	0.003	1.1	0.3	3.4	0.916	2.6	0.6	10.5	0.186
Injected on average >3 times per day in the past 30 days (vs. <2 times/day)		1.1	0.4	2.9	0.805	4.5	1.6	12.8	0.005
Shared syringes in the past 12 months (vs. No syringe sharing in the past 12 months)	0.002	2.5	1.2	5.2	0.014	4.7	1.9	11.5	0.001
No condom use in last intercourse (vs. Use of condom or non-active)	0.043	0.8	0.5	1.4	0.424	0.4	0.2	0.9	0.018
Never tested for HCV before (vs. Tested for HCV before)	0.001	0.6	0.3	0.9	0.028	0.2	0.1	0.5	<0.001

^a^Diagnosed HCV positive and HIV negative. ^b^Diagnosed HCV positive and HIV positive. ^c^Diagnosed HCV negative and HIV negative. ^d^Overall *p*-value for this covariate. ^e^RRR: relative risk ratios. ^f^95 % confidence intervals. ^g^Gender was measured by the following item: “What is your gender?” Response options included “male”, “female”, “other (transgender)”. No person responded “other” in the sample used in the present analysis. ^h^Includes children and / or parents

Risk factors for HCV/HIV coinfection, but not HCV monoinfection, were residence in major urban areas (RRR = 4.8, 95 % CI: 1.7–13.7, *p* = 0.004) and averaging > 3 injections a day in the last 30 days (RRR = 4.5, CI: 1.6–12.8, *p* = 0.005). The risk of coinfection was reduced in injection drug users who did not use a condom in the last sexual intercourse (RRR = 0.4, CI: 0.2–0.9, *p* = 0.018).

Additional multivariate analyses, with the HCV monoinfected group as the reference category, showed that the risk of HCV/HIV coinfection was higher among those living in urban areas (RRR = 3.4, 95 % CI: 1.3–8.7, *p* = 0.012), averaging >3 injections a day in the last 30 days (RRR = 4.0, CI: 2.0–8.2, *p* < 0.001), and lower among injection drug users who were aged ≥35 years (RRR = 0.4, CI: 0.2–0.7, *p* = 0.004), did not use a condom in the last sexual intercourse (RRR = 0.5, CI: 0.3–0.9, *p* = 0.030), and had never previously been tested for HCV (RRR = 0.4, CI: 0.2–0.8, *p* = 0.015) (data not shown in Table).

## Discussion

Greece is a country with a high HIV and HCV epidemic among people who inject drugs (41, 46). In the present study we estimated the prevalence of HCV/HIV coinfection in a sample of injection drug users entering OST in Greece in 2013 to be 14.9 %, with almost all HIV seropositive persons (95.3 %) having HCV comorbidity. We also aimed to identify injection drug users at the greatest risk of HCV/HIV coinfection on the basis of their socio-demographic, drug use and higher-risk behavioral characteristics. Our findings corroborate existing evidence suggesting that lengthy injecting careers, syringe sharing and prior incarceration independently increase the risk of infection. The present study additionally showed that the risk also increases in people who inject drugs and live alone or with a spouse/partner without children (as opposed to living with parents and /or children). Risk factors for HCV/HIV coinfection, but not HCV monoinfection, were residence in major urban areas and averaging more than 3 injections a day in the past 30 days.

Specifically, injection drug users with injecting histories of 10 or more years were at increased risk for both HCV monoinfection and HCV-HIV coinfection (almost six and fourteen times higher, respectively) compared to drug users with shorter injecting histories. Similarly, sharing a used syringe also increased the risk of infection, with those who reported syringe sharing in the 12 months preceding the treatment entry having almost five times greater risk of coinfection compared to those who had not shared in the past year. These findings are supported by ample evidence implicating long injecting histories and syringe sharing as key risk factors for coinfection [31–33, 35, 51]. There is also ample evidence suggesting that interventions that offer OST and HCV and HIV treatment to infected persons, coupled with needle and syringe programs (NSPs) and safer injecting rooms, may be vital in interrupting higher-risk injecting routines and reducing syringe sharing, thereby preventing new infections and reducing prevalence in this population (see [52] for review; also [53–60]). Harm reduction programs in Greece have generally had limited coverage [61, 62]. The 2011 HIV outbreak in injection drug users, coupled with international pressure and EU funding support, led to a public health response that focused on enforcement-based interventions covering diagnosis, scaling-up NSPs and OST, and linkage of HIV seropositives to ART (see e.g., [46]). Although restricted to Athens, interventions coincided with a significant reduction of HIV incidence [46, 63]. However, these programs have not proved financially sustainable [64]. Furthermore, under the austerity policies imposed from 2010 up to the present, public spending allotted to harm reduction has been minimal.

Living alone or only with a spouse/partner (without children or other family members, e.g., parents) trebled in our study the risk of HCV infection with or without HIV. Previous research has shown that higher-risk drug use is positively associated with intimacy among friendship networks [65]. At the same time, pockets of social support may reduce HCV and HIV related morbidity and mortality [66]. Taking care of children and living with a member of one’s (biological) family is an often overlooked yet potentially important aspect of social support, especially in countries like Greece where the family retains its pivotal role in the lives of drug users [67]. The latter may influence health behaviors through self-regulation in conformity to norms or through others’ health promoting behaviors and expectations [68]. In our study the likely absence of social support may have nurtured the adoption of risky behaviors in relation to HIV.

Our findings also suggest that those who have been incarcerated have twice as high a risk of HCV infection with or without HIV, suggesting that prison environments foster high-risk injection and sexual behavior and hence the acquisition of infection. The cross-sectional nature of our data does not allow us to make inferences about causality, but the independent association between incarceration and HCV, HIV, and HCV/HIV coinfection in this population is well documented [51, 69]. In Greece, no administrative alternatives to imprisonment are implemented for drug users. As of 2016, prisoners in Greece do not have access to sterile injection equipment or condoms, while screening, HIV counseling, and ART are not routinely available [70]. Since 2015, OST and drug-related health care has been available in only two of the eighteen prison units in operation in the country. Furthermore, following incarceration, low coverage of services and other structural barriers (e.g., lack of essential documents, language restrictions, poverty, stigma or fear) may have promoted behaviors with higher health risk in this population.

HCV/HIV coinfection (but not HCV monoinfection) was independently associated with frequent injection and residing in metropolitan areas. More specifically, living in an urban area and averaging more than 3 injections a day increased the risk by almost five. Living in an urban area represents a marker for high-risk factors. These factors include barriers in access to health services coupled with relatively easy access to multiple and novel substances of unknown composition and adverse effects, participation in larger, unknown and changing injection and sexual networks, and exposure to sex work (e.g. [5]). In addition, fear of arrest or punishment may displace or lead drug injectors to avoid using outreach programs, or to hurried injections and injection practices that increase opportunities for parenteral exposures to HIV.

Theoretically, having a test for infectious diseases is an indicator of health-protecting attitude [50], leading to the assumption that drug users who have been tested in the past will more likely be uninfected. Counterintuitively, our study showed that the risk of being infected was negatively associated with previous HCV testing. Unfortunately, the cross-sectional nature of the present study and the fact that we did not measure the time at which both the infection and previous testing occurred, or whether the test result was known to the participant, prevent us from being able to interpret these findings.

There was also a negative association between having used a condom in the last sexual intercourse and the risk of HCV/HIV coinfection (but not of HCV monoinfection). Again, lack of additional data (e.g., frequency of sexual activity, partner’s sexual orientation etc.) or more relevant variables (e.g., overall consistency of condom use) prevent us from fully explaining these findings. However, a number of hypotheses may be proposed: first, it may be that those who responded that they *did* use a condom in their last sexual intercourse had been –prior to the last occasion– largely reckless in their sexual behavior. Second, the category included people who had no sex in the last 12 months, who possibly knew their positive serological status and abstained from sex. Third, the present data were collected in a period in which the HIV epidemic and the corresponding health-risk awareness and HIV counselling programs were at their greatest extent, reaching a substantial number of injection drug users (especially in Athens) [41, 46, 62]. Against this background it may be that those who reported that they *did* use a condom in the last sexual intercourse were giving the socially desired response. Finally, it may also be that those who responded that they *did not* use a condom in the last sexual intercourse were more cautious with other high-risk behaviors.

The findings of our study should be seen in the light of several limitations. First, the study participants were recruited only from OST outpatient clinics and only from central and southern Greece. In theory this affects the generalizability of our findings. However, clinics and participants in the present study comprised about 70 % of national totals that year (2013). Second, we focused only on injection drug users and injection practices in general among heroin and other opioid users. Using stimulants (e.g., in the case of Greece, ‘shisha’, a variant of methamphetamine) also has destabilizing effects [5, 71]. However, only a small proportion of study participants reported primary use of stimulants, including cocaine (2.4 %), and therefore this behavior was not included as a correlate in the analysis. Third, the cross-sectional design of the study does not allow the establishment of a causal relationship or a direction of causality between empirically related variables. Furthermore, as we did not measure whether HIV and HCV seropositives were known positives prior to the data collection, or whether they had already received specialised infectious diseases treatment, we could not check for possible interactions with other variables examined in the study. Finally, although data collection protocols have been employed for years in ways that elicit valid responses, possible misreporting associated with recall and social desirability biases [72] during the interview cannot be overlooked.

## Conclusions

The positive association observed between infection and lengthy injection histories points to the need for scaling-up OST programmes and retaining people in treatment. The positive association between HCV/HIV coinfection and, independently, frequent daily injection and living in urban areas suggests that more injection equipment, screening and brief advice are required in order to reach out to more injection drug users, especially in the inner city areas of all major cities in the country. Prior incarceration increases the risk of infection, and this alone highlights the need to provide routine testing and harm reduction services in all detention centres in the country. Importantly, keeping in contact with their family (i.e., parents and/or children) may reduce the risk of infection for people who inject drugs, and this element could guide counselling that takes place at the treatment centre level and relies on building up family support systems.

## Abbreviations

AIDS: Acquired immunodeficiency syndrome
ART: Antiretroviral therapy
CI: Confidence interval
EFTA: European Free Trade Association
EIA: Enzyme immunoassay
ELISA: Enzyme-linked immunosorbent assay
EMCDDA: European Monitoring Centre for Drugs and Drug Addiction
EU: European Union
HCV: Hepatitis C virus
HIV: Human immunodeficiency virus
NSP: Needle and syringe programs
OKANA: Greek Organization Against Drugs
OST: Opioid substitution treatment
PWID: People who inject drugs
RIBA: Recombinant immunoblot assay
RNA: Ribonucleic acid
RRR: Relative risk ratio
